# Variations of Histone Modification Patterns: Contributions of Inter-plant Variability and Technical Factors

**DOI:** 10.3389/fpls.2017.02084

**Published:** 2017-12-07

**Authors:** Sylva Brabencová, Ivana Ihnatová, David Potěšil, Miloslava Fojtová, Jiří Fajkus, Zbyněk Zdráhal, Gabriela Lochmanová

**Affiliations:** ^1^Mendel Centre for Plant Genomics and Proteomics, Central European Institute of Technology, Masaryk University, Brno, Czechia; ^2^Laboratory of Functional Genomics and Proteomics, National Centre for Biomolecular Research, Faculty of Science, Masaryk University, Brno, Czechia

**Keywords:** *Arabidopsis thaliana*, ecotype, histone, post-translational modifications, epigenetics, mass spectrometry

## Abstract

Inter-individual variability of conspecific plants is governed by differences in their genetically determined growth and development traits, environmental conditions, and adaptive responses under epigenetic control involving histone post-translational modifications. The apparent variability in histone modifications among plants might be increased by technical variation introduced in sample processing during epigenetic analyses. Thus, to detect true variations in epigenetic histone patterns associated with given factors, the basal variability among samples that is not associated with them must be estimated. To improve knowledge of relative contribution of biological and technical variation, mass spectrometry was used to examine histone modification patterns (acetylation and methylation) among *Arabidopsis thaliana* plants of ecotypes Columbia 0 (Col-0) and Wassilewskija (Ws) homogenized by two techniques (grinding in a cryomill or with a mortar and pestle). We found little difference in histone modification profiles between the ecotypes. However, in comparison of the biological and technical components of variability, we found consistently higher inter-individual variability in histone mark levels among Ws plants than among Col-0 plants (grown from seeds collected either from single plants or sets of plants). Thus, more replicates of Ws would be needed for rigorous analysis of epigenetic marks. Regarding technical variability, the cryomill introduced detectably more heterogeneity in the data than the mortar and pestle treatment, but mass spectrometric analyses had minor apparent effects. Our study shows that it is essential to consider inter-sample variance and estimate suitable numbers of biological replicates for statistical analysis for each studied organism when investigating changes in epigenetic histone profiles.

## Introduction

Epigenetic traits are heritable traits that are not linked to changes in DNA sequence. In a narrower sense, they involve spatiotemporal changes in gene activity realized mainly via modifications of major chromatin components – methylation of cytosines in DNA and modifications of histone proteins. These mechanisms, together with the activity of small and non-coding RNA molecules and distribution of histone variants, determine the structure of the chromatin in affected regions and subsequently influence crucial cellular processes, e.g., gene expression, DNA repair and replication timing. In this respect, chromatin is the natural substrate for all DNA-targeted processes. Epigenetics is also regarded as a major link between genotype and phenotype, as it is involved in the regulation of developmental processes, cell differentiation and adaptation to suboptimal living conditions.

Historically, and rather simplistically, chromatin was divided into condensed heterochromatin (consisting predominantly of repetitive sequences, silenced transposons and repressed genes) and open euchromatin, encompassing actively transcribed genes. Following further analyses, functionally distinct chromatin regions associated with specific patterns of histone modifications have been defined. For example, five and subsequently nine types of chromatin “colors” have been described in Drosophila, based on the genetic characterization of 53 chromatin proteins (Filion et al., 2010), distributions of epigenetic histone marks, and associated variations in factors such as DNaseI sensitivity, binding of non-histone proteins and transcription levels (Kharchenko et al., 2011). In the model plant *Arabidopsis thaliana*, Roudier et al. (2011) distinguished four chromatin states based on the distribution of 11 histone modifications and level of DNA methylation: transcriptionally active genes characterized by the presence of trimethylated H3K4 and H3K36; two types of repressive chromatin – regions associated with H3K27me3 under polycomb group-mediated control and classical heterochromatin marked by H3K9me2 and H4K20me1; and finally weakly expressed genes and intergenic regions with no typical set of chromatin modifications. In a subsequent more detailed study considering 16 chromatin features—including DNA sequence motifs, nucleosome occupancy, cytosine methylation, histone variants and histone modifications—Sequeira-Mendes et al. (2014) characterized nine functional chromatin states in *A. thaliana*. They also found distinct preferential associations of these chromatin states, further increasing the complexity of the plant’s chromatin arrangements.

The distribution of epigenetic marks in the chromatin of mammalian cells has been characterized in similar analyses, for instance in the ENCODE Project (Birney et al., 2007).

Thus, there are species- and locus-specific combinatorial patterns of epigenetic modifications, and analysis of the epigenome in cells or tissues in specific developmental stages or subjected to different conditions may be essential for understanding both complex epigenetic responses and the modulation of individual epigenetic marks. This is important for diverse applications as epigenetic biomarkers are used (for example) in clinical diagnosis, development of modern therapeutic approaches as well as in studies of plants’ responses and adaptation to global environmental changes. The traditional approach for analyzing histone modifications and histone variants involves chromatin immunoprecipitation using antibodies recognizing specific modifications followed by high-throughput analysis of immuno-precipitated fractions (ChIP-seq or ChIP-on-chip). Given the limitations of antibody-based approaches (e.g., cross-reactivity and limited epitope accessibility), mass spectrometry (MS) – assisted approach providing highly standardized procedures for sample preparation, high resolution instrumentation and data analysis algorithms, is currently an important analytical tool to study post-translational modifications (PTMs; Aebersold and Mann, 2016). It has been extensively used for both qualitative and quantitative analyses of histone modifications, including phosphorylation, methylation and acetylation in many model species (Drury et al., 2012; Tweedie-Cullen et al., 2012; Krejčí et al., 2015; Moraes et al., 2015; de Jesus et al., 2016; Henry et al., 2016), as reviewed by Zheng et al. (2016). MS also offers the ability to examine novel and combinatorial PTMs in a high-throughput fashion. Direct “bottom–up” analysis, the most commonly used MS-based strategy, has limited utility for detecting combinatorial histone PTMs due to the shortness of peptides obtained by the commonly used trypsin digestion. However, this issue can be partially solved by chemical derivatization of lysines prior to enzymatic cleavage, allowing the correlation of modifications on multiple aminoacid residues within the same sequence region (Plazas-Mayorca et al., 2009).

Whatever instruments are used, high reproducibility, accurate quantitative results, and thus robust experimental designs are clearly essential in any statistical analysis. In a reliable differential proteomic study, statistical power of the applied test should accompany traditional metrics (fold-change, *p*-value) (Levin, 2011). Both, *p*-value and statistical power depend on the variability and the number of replicates. Thus, the numbers of biological and technical replicates included in the analysis as well as determination of natural and technological sources of inter-individual variations in investigated proteins are crucial (Levin, 2011; Al Shweiki et al., 2017).

In the study presented here, we examined the inter-individual variability of histone mark levels in samples of independently cultivated plants to assess the numbers of replicates required for rigorous evaluation of changes in epigenetic histone modification patterns. Using mass spectrometry, we analyzed PTMs of H3 and H4 histones in *A. thaliana* plants of two frequently used ecotypes, Columbia 0 (Col-0) and Wassilewskija (Ws). Plants grown from seeds collected from a single parent plant or progenies of a set of parent plants (designated Single and Mixed samples, respectively) were compared. We also compared effects of two plant tissue homogenization techniques (grinding in a cryomill or with a mortar and pestle) on protein contents and proportions of histone proteins in the extracts. The acquired data should facilitate selection of appropriate experimental design for analyses of plants’ complex histone modifications.

## Materials and Methods

### Plant Material and Growth Conditions

*Arabidopsis thaliana* (ecotypes Col-0 and Ws) seeds were collected (i) from one plant (Single sample; Supplementary Figure 1A) or (ii) from several parent plants cultivated at the same time (Mixed sample; Supplementary Figure 1A; these plants were progenies of plants cultivated at different times). Seeds were sterilized by ethanol and sown on half-strength Murashige-Skoog medium (Duchefa Biochemicals) agar plates. The seeds were germinated in phytotrons under short day conditions (8 h light, illumination 100 μmol.m^-2^.s^-1^ at 21°C and 16 h dark at 19°C) to favor leaf production (Mouradov et al., 2002). Seven-day old seedlings were transferred to soil and plants were grown under short day conditions in the phytotrons. Five to seven leaves in good shape were harvested from the 6 – 7-week old plants (before bolting), rinsed with sterile water, dried and frozen.

### Nuclei Isolation and Histone Extraction

For the investigation of histone peptide levels variability within the ecotypes, sampled leaves (ca. 300–500 mg) of *A. thaliana* (ecotypes Col-0 and Ws) were ground in liquid nitrogen using mortar and pestle. For comparison of homogenization procedures, leaves of *A. thaliana* (ecotype Col-0; ca. 300–500 mg) were ground in liquid nitrogen using a mortar and pestle, or a Spex SamplePrep 6870 Freezer/Mill^®^ cryomill (2 × 2.5 min grinding separated by 2 min cooling). Following homogenization, samples were resuspended in an extraction buffer consisting of 10 mM NaCl, 10 mM 2-(*N*-morpholino) ethanesulfonate (pH 6.0), 5 mM EDTA, 0.25 M sucrose, 0.6% Triton X-100, 0.2 M spermidine, 100 μM PMSF and 20 mM β-mercaptoethanol to “soft ice” consistency. The homogenate was filtered through nylon mesh and centrifuged (10 min, 3000 *g*, 4°C). The pellet was washed twice with the extraction buffer, resuspended in Percoll buffer (2.4 g of 5× concentrated extraction buffer, 18 g of Percoll from Sigma–Aldrich) and centrifuged (15 min, 4000 *g*, 4°C). Nuclei floating on the Percoll buffer surface were collected and subjected to three cycles of resuspension in washing buffer (75 mM NaCl, 10 mM EDTA, 50 mM Tris-HCl pH 8.0) and collection by centrifugation (10 min, 3000 *g*, 4°C). They were then resuspended in CHAPS buffer (50 mM Tris-HCl pH 8.0, 100 mM NaCl, 3 mM EDTA, 1% CHAPS, 0.1 μM PMSF, 45 mM sodium butyrate, and 10 μl/ml of P9599 protease inhibitor cocktail from Sigma–Aldrich), incubated for 1 h on ice, and centrifuged (8 min, 10,000 *g*, 4°C). Finally, the pellets were resuspended in 200 – 400 μl of ice-cold 0.2 M H_2_SO_4_ and incubated overnight, with shaking at 4°C, the samples were centrifuged (8 min, 16,100 *g*, 4°C) and supernatants containing histone proteins were collected.

### Histone Sample Preparation for Mass Spectrometry

Whole volume of histone extracts was processed using the filter-aided sample preparation (FASP) procedure described by Wiśniewski et al. (2009); the protein concentration measurement was omitted based on previously proven reproducibility of sample preparation before FASP (Supplementary Table 2). Briefly, proteins dissolved in H_2_SO_4_ were mixed with urea buffer (8 M urea, 0.1 M Tris-HCl pH 8.5) in a 1:3 ratio, placed in a YM-10 Microcon filter unit (Millipore) and centrifuged (40 min, 14,000 *g*, 20°C). After three washes with urea buffer, proteins in the samples were reduced and alkylated by adding 0.1 mM dithiothreitol and 0.05 M iodoacetamide, respectively, to the buffer then the samples were centrifuged. The samples were washed with urea buffer, then with 50 mM ammonium bicarbonate pH 8.0 (ABC) and digested overnight at 37°C with sequencing grade modified trypsin (Promega) in 50 μl of 50 mM ABC (500 ng of trypsin per sample). The digests were collected by centrifugation (15 min, 14,000 *g*, 20°C) then washed twice more with 50 μl of 50 mM ABC. The peptide concentration was determined using a Micro BCA^TM^ Protein Assay Kit (Thermo Fisher Scientific).

In the experiments including comparison of the homogenization methods’ effects, histone derivatization by propionic anhydride, essentially following Sidoli et al. (2016), was performed. Sulfuric extracts (prepared as described above) containing 16 μg of plant histone proteins were placed in YM-10 Microcon filter units (Millipore). For protein concentration measurement, aliquots of sulfuric extracts were sixteen times diluted with deionized water and protein content was determined using a Micro BCA^TM^ Protein Assay Kit. Histone samples were subjected to a double round of propionic anhydride derivatization at both protein and peptide levels. The samples were concentrated in a Savant SPD121P SpeedVac concentrator (Thermo Fisher Scientific) to 3 μl and diluted with 0.1% FA to a volume of 100 μl. Labeled histones were desalted using a Hypersep SpinTip C-18 column (Thermo Fisher Scientific), and the peptide concentration was determined using a Micro BCA^TM^ Protein Assay Kit.

### Mass Spectrometric Analysis

Tryptic digests of each plant group represented by eight biological replicates (histone samples isolated from eight individual plants of the respective ecotype) were measured in three technical replicates using liquid chromatography-tandem mass spectrometry (LC-MS/MS) to enable statistical evaluation of variations in levels of histone peptide-forms (Supplementary Figure 1A). The samples were spiked with the iRT-C18 reference peptides (RT-Kit; Biognosys). The LC-MS/MS equipment consisted of an RSLCnano system, equipped with an Acclaim Pepmap100 C18 analytical column (3 μm particles, 75 μm × 500 mm; Thermo Fisher Scientific), coupled to an Orbitrap Elite hybrid spectrometer (Thermo Fisher Scientific) equipped with a Digital PicoView 550 ion source (New Objective) using PicoTip SilicaTip emitter (FS360-20-15-N-20-C12), and Active Background Ion Reduction Device. The mobile phase consisted of 0.1% formic acid in water (A) and 0.1% formic acid in 80% acetonitrile (B), with the following proportions of B: 1% for 5 min at 500 nl/min to concentrate peptides, then (with a switch to 300 nl/min) 1–13% over 20 min, 13–33% over 25 min, 33–56% over 20 min and 56–80% over 5 min followed by isocratic washing at 80% B for 8 min. The analytical column was re-equilibrated with 99:1 (mobile phase A:B) prior to loading the next sample in the sample loop. The analytical column outlet was directly connected to the ion source of the MS. MS data were acquired using a data-dependent strategy selecting up to top 10 precursors based on precursor abundance in a survey scan (350–2000 m/z). The resolution of the survey scan was 60,000 (400 m/z) with a target value of 1 × 10^6^, one microscan and maximum injection time of 1000 ms. HCD MS/MS spectra were acquired with a target value of 50,000 and resolution of 15,000 (400 m/z). The maximum injection time for MS/MS was 500 ms. Dynamic exclusion was enabled for 45 s after one MS/MS spectrum acquisition and early expiration was disabled. The isolation window for MS/MS fragmentation was set to 2 m/z.

For analysis of derivatized samples (represented by three biological replicates for each group, Supplementary Figure 1B), the same instrument and experimental conditions were used for LC-MS/MS but the gradient was modified due to the higher hydrophobicity of derivatized peptides: following the peptide concentration step, the percentage of B was linearly increased from 1 to 70% over 90 min, then to 85% over 20 min, and this percentage was held for 10 min.

### Database Searches and Quantification of Histone Peptide Forms

The RAW mass spectrometric data files were analyzed using Proteome Discoverer software (Thermo Fisher Scientific; version 1.4) with in-house Mascot search engine (Matrixscience, version 2.6) to compare acquired spectra with entries in the UniProtKB *Arabidopsis thaliana* protein database (version 2017_01; 27332 protein sequences ^1^), cRAP contaminant database^2^ and in-house histone database (version 2017_02; 71 protein sequences). Mass tolerances for peptides and MS/MS fragments were 7 ppm and 0.025 Da, respectively. For the histone database searches, no enzyme specificity and the following modifications were set: oxidation (M), deamidation (N, Q), acetylation (K, protein N-term), methylation (K, R), dimethylation (K), trimethylation (K) and phosphorylation (S, T) as variable modifications and carbamidomethylation (C) as a static modification. For searches against the cRAP and UniProtKB *Arabidopsis thaliana* databases, trypsin enzyme specificity with up to three missed enzyme cleavages and the following modifications were set: oxidation (M), deamidation (N, Q), acetylation (K, protein N-term) as variable peptide modifications and carbamidomethylation (C) as a static modification. Rank 1 peptides with Mascot expectation value < 0.01 and at least six amino acids were considered. Log10-transformed histone protein areas were summed in individual samples to prove that comparable histone pools were obtained across biological replicates (Supplementary Figure 2). Peptide identifications were manually verified and quantitative data were evaluated using Skyline 3.6 software.

For derivatized samples, semi-Arg-C for enzyme specificity allowing up to two missed cleavages was set. For searches against cRAP and UniProtKB *Arabidopsis thaliana* databases the variable modification settings were oxidation (M), deamidation (N, Q), acetylation (K, protein N-term) and propionylation (K, protein N-term), while for histone database searches they were acetylation (K, protein N-term), methylation (K, R), dimethylation (K), trimethylation (K), phosphorylation (S, T), and propionylation (K, protein N-term, S, T, Y). The abundance of histone peptides was quantified automatically using Proteome Discoverer 1.4 software. The peak area corresponding to each precursor ion was calculated from the extracted ion chromatograms (XICs) using the Precursor Ions Area Detector node. Selected histone peptide identifications were manually verified and quantified from the peak areas derived from the XICs using Skyline 3.6 software, including identification alignment across the raw files based on retention time and m/z. The relative abundance (RA) of specific modified histone forms was calculated according to the following formula [25]:

$$\mathrm{RA}=\frac{\Sigma \,\;\mathrm{peak areas of XICs of peptides with certain PTM}}{\Sigma \,\;\mathrm{peak areas of XICs of all forms of the peptide}}.$$

### Data Analysis

To enable statistical evaluation of levels of histone modifications in the *A. thaliana* ecotypes and their inter-individual variability, areas of peptide precursors in the XICs were normalized to areas of unmodified peptides of the corresponding histones and log2-transformed. The Grubbs test for outliers was applied and detected outliers were removed. Using the Real Statistics Resource Pack for MS Excel and R (R Core Team, 2017), both linear mixed models (LMMs) and F-tests were applied to assess inter-individual variability. First, the lme4 package (Bates et al., 2015) was used to fit an LMM with no fixed-effect and biological and technical replicates as random effects to data on levels of each peptide. Biological and technical variabilities estimated from the model were subsequently used to calculate the number of replicates needed to detect selected fold-changes (1.5, 1.75, and 2) by a hierarchical model with a minimum statistical power of 0.8 and significance threshold of 0.05, as recommended by Trutschel et al. (2015). Second, peak areas of each peptide in technical replicates were averaged and variances were compared by *F*-tests (significance threshold α = 0.01). Resulting *p*-values were combined following Fisher (1925). Effects of the two sample homogenization techniques on peptide levels and representation of histone peptides in the samples were evaluated as follows. The mean and standard deviation of the log2-transformed abundance of each peptide observed in each group of plants subjected to each homogenization technique were calculated and compared by Mann–Whitney tests and Spearman’s correlation analysis.

## Results

### High Inter-individual Variability of Histone Mark Levels in *A. thaliana* Ecotype Ws

To assess the inter-individual variability of histone mark levels in our *A. thaliana* plants, the abundance of selected histone H3 and H4 post-translationally modified peptides in Single and Mixed samples (as described above) of both ecotypes was evaluated. Histone extracts from leaves of eight independently cultivated plants of each group were analyzed, in random order in three technical batches by MS, so there were 24 replicates in total per group. This enabled estimation not only of biological variability within each group but also the technical variability introduced by LC-MS/MS analysis (illustrated in Supplementary Figure 1A).

**FIGURE 1:**
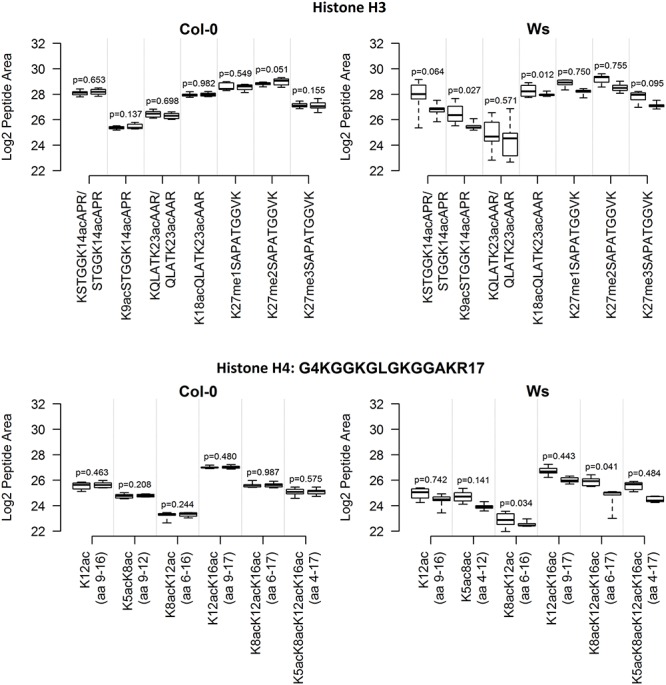
Inter-plant variability of selected histone mark levels in *A. thaliana* Col-0 and Ws ecotypes grown from seeds collected from a single parent plant (Single) and a set of parent plants (Mixed), after aggregation of technical replicates. For each ecotype and histone, the distribution of normalized log2-transformed peptide precursor XIC peak areas is represented by a pair of boxplots: Mixed **(left)** and Single **(right)**. Abundances of peptides with missed enzyme cleavages (e.g., QLATKAAR and KQLATKAAR) were summed. The boxplots show extremes, interquartile ranges and medians obtained from analyses of samples of eight plants (averages of three technical batches). Differences in variances in abundances of individual peptides between Mixed and Single samples were compared by *F*-tests, *p*-values are indicated.

In total, seven and six selected peptide-forms from H3 and H4 histones, respectively, were quantified in Skyline software. The first technical batch of Ws Single samples was accidentally excluded from data evaluation due to emitter failure during LC-MS/MS analyses. The log2-transformed precursor areas of all biological replicates (following removal of outliers detected by the Grubbs test and averaging technical replicates) are shown in **Figure 1** (see Supplementary Table 1 for descriptive statistics). The most frequent epigenetic modification detected was acetylation. For example, four acetylated lysines were detected in an H4 peptide comprising 14 amino acids, and shorter variants with one, two and three acetylated lysine(s) were also detected. In H3 peptides, the pattern of epigenetic modifications was less complex in terms of numbers of marks per peptide (two acetylated lysines in two peptides), but peptides with mono-, di- or tri-methylated lysine were observed. There was no significant (*p* < 0.01) difference in variance of the abundance of particular peptides between Single and Mixed samples, of either Col-0 or Ws ecotypes. However, when *p*-values for selected peptides were combined, there was a significant difference in variance between Single and Mixed Ws samples (*p* < 0.001), but not in Col-0 samples (*p* = 0.432). Thus, levels and variations of modified histone peptides were similar in Single and Mixed samples of Col-0 ecotype, but inter-individual variability was significantly higher in Mixed samples than in Single samples of ecotype Ws.

**FIGURE 2:**
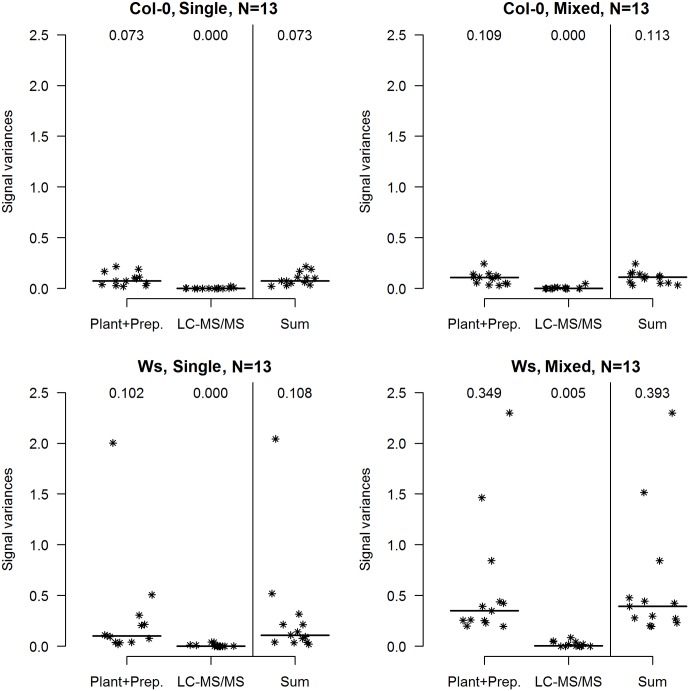
Contributions of biological and technical components to the total variance of observed histone mark levels in *A. thaliana* Col-0 and Ws ecotypes grown from seeds collected from a single parent plant (Single) and a set of parent plants (Mixed). In each case, the total variance (Sum) of the abundance of selected individual H3 and H4 histone peptide forms (*N* = 13) was decomposed into variance related to plant cultivation and sample preparation (biological, denoted Plant + Prep.) and variance related to measurement by the LC-MS/MS system (technical, denoted LC-MS/MS) by LMM. Medians are indicated by horizontal bars and numbers.

The inter-individual variability related to the sampled plants’ homogeneity was further examined using LMM, by decomposing the total observed variance into a component related to plant cultivation and sample preparation (biological replicates) and a technical component related solely to multiple injection by LC-MS/MS (technical replicates). The experiment was not designed to further separate the purely biological component from a sample preparation component as amounts of starting material were too low to divide the samples before nuclei isolation (median fresh weights of leaves from the Col-0 and Ws plants were ∼ 0.5 g and 0.3 g, respectively). The median sum of variances of all random effects was higher in Mixed than in Single samples of both ecotypes, but the difference was only significant for Ws samples (**Figure 2**; *p* = 0.006, Mann–Whitney test). As expected, technical variability related to individual LC-MS/MS experiments made minimal contributions to the total variability. In data obtained from analyses of both ecotypes, the variance of the random effect of repeated measurements was close to 0 for most of the peptides and both sample types (**Figure 2**). To assess the loss of reproducibility due to emitter failure we also calculated technical variance from the data including the first technical batch of Ws Single samples (Supplementary Figure 3).

### More Replicates Are Needed for Histone Modification Analysis of *A. thaliana* Ecotype Ws

Based on the technical and biological variability estimated by LMMs, the number of replicates of both types of samples (Single and Mixed), of both ecotypes, required for reliable analysis of prospective differences in histone modification levels was assessed. The minimum number of replicates needed for detecting peptides with a minimal mean difference in log2-transformed peptide precursor areas of 0.58, 0.8, and 1 (corresponding to 1.5-, 1.75- and 2- fold changes, respectively) by a hierarchical model with statistical power of 0.8 and significance threshold *p* < 0.05 was similar for Single and Mixed samples of Col-0 (**Table 1**). However, more replicates were needed for corresponding analyses of Ws plants grown from seeds of either a mixture of individuals or a single individual, especially to detect peptides with <1.75-fold changes in abundance (**Table 1**). These data further highlight the need to consider the level of inter-individual variability and its differences among *A. thaliana* ecotypes in proteomic-based epigenetic analyses.

**Table 1:** Required numbers of replicates for proteomic study of *A. thaliana* Col-0 and Ws ecotypes grown from seeds collected from a single parent plant (Single) and a set of parent plants (Mixed).

	Kind of replicate	Fold-change				
		1.5		1.75		2
**Col-0**						
Mixed	Biological	7	6	4		3
	Technical	1	2	1		1
Single	Biological	6		4		3
	Technical	1		1		1
**Ws**						
Mixed	Biological	30	29	16		11
	Technical	1	2	1		1
Single	Biological	15		9	8	6
	Technical	1		1	10	1

*Numbers of plants and repeated analyses of each plant using LC-MS/MS needed to detect 1.5-, 1.75-, and 2-fold changes in protein abundance with 95% confidence and 80% power are shown*.

### Manual Homogenization of Plant Tissues Is More Suitable than Cryomilling for Preparing Histone Extracts for Mass Spectrometry Analysis

**FIGURE 3:**
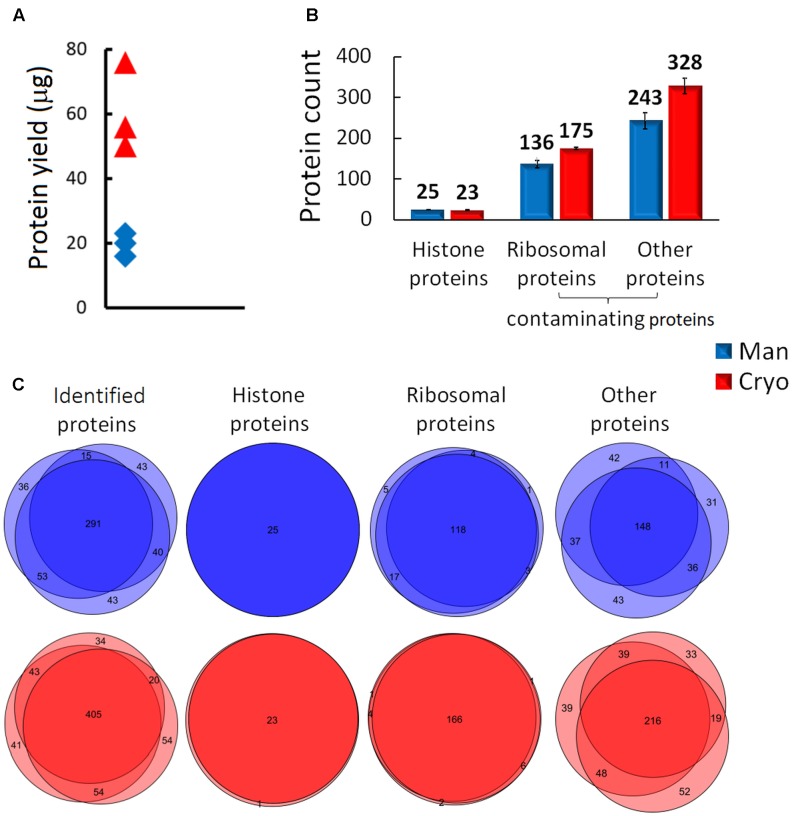
Comparison of the quality of histone protein extracts obtained by manual plant tissue homogenization (Man) and using a cryomill (Cryo). **(A)** Differences in protein yield, **(B)** distributions of identified proteins (means and SD, *N* = 3) and **(C)** Venn diagrams displaying the number of overlapping protein groups identified in biological triplicates.

**FIGURE 4:**
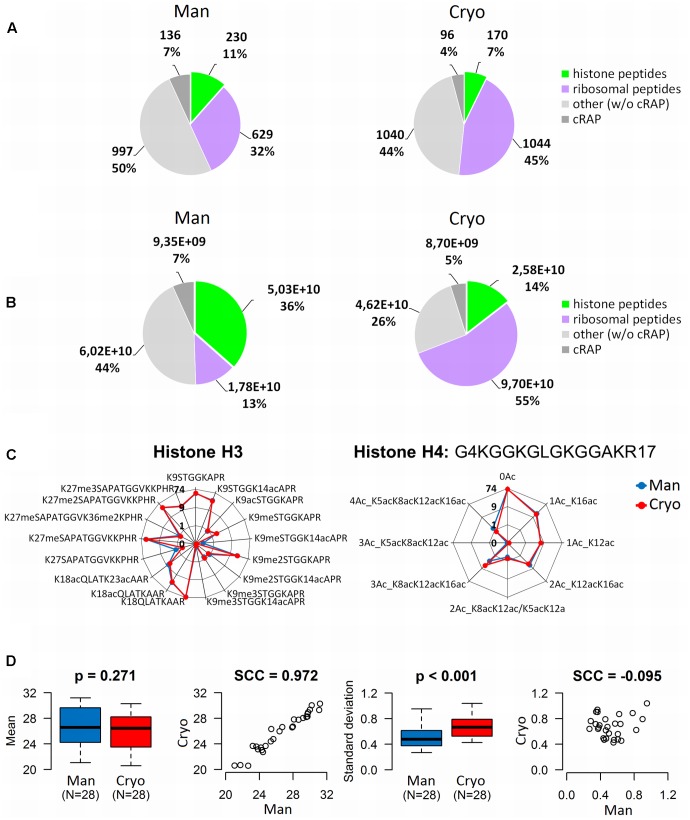
Comparison of the quality of histone peptides obtained by manual plant tissue homogenization (Man) and using a cryomill (Cryo). Differences in qualitative **(A)** and quantitative **(B)** distributions of identified peptides (merged data, *N* = 3). **(C)** Radar charts showing RAs of histone H3 and H4 peptide forms (ratios of XIC peak areas of peptides with given PTMs to the sum of peak areas of all forms of H3 or H4 peptides, respectively, in percentages). *Y*-values are binary logarithms, with zero at the center of each chart. **(D)** Box-plots and scatter-plots of the means and standard deviations of abundances of histone H3 and H4 peptide forms detected in the samples. The boxplots show extremes, interquartile ranges and medians (*N* = 28). Means and standard deviations were compared by Mann–Whitney tests (*p*-values) and calculating Spearman’s correlation coefficients (SCC values).

The importance of choosing an appropriate sample processing method for proteomic histone analysis was demonstrated by comparing the quality of extracts of Mixed samples (biological triplicates of leaves of the Col-0 ecotype) generated by manual homogenization (with a mortar and pestle) and cryomilling. In both cases the recovery of target molecules was increased by propionylation of lysine residues, which allows production of longer and more hydrophobic peptides, thereby improving the characterization and quantification of histone peptide forms by LC-MS (Sidoli et al., 2016). The data acquired from subsequent MS analysis (the full experimental design is shown in Supplementary Figure 1B) were evaluated using Proteome Discoverer software. Cryomilling resulted in higher total protein yields than the manual treatment (**Figure 3A**) which correlated with higher numbers of identified proteins using LC-MS/MS analysis (**Figure 3B**). But, unfortunately it released higher numbers of contaminating proteins: 503 ± 21 and 379 ± 20 were detected in cryomilled and manually ground samples, respectively (**Figure 3B**). We observed higher reproducibility of protein identifications within the replicates of cryomilled samples compared to manual grinding. On the other hand, identified histone proteins entirely overlapped among the replicates of both homogenization methods (**Figure 3C**). In addition, the proportion of histone peptides was unexpectedly low in cryomilled samples: 170 unique histone peptides compared to 230 in manually ground samples, representing 7 and 11% of the total number of detected peptides, respectively (**Figure 4A**).

There was a corresponding difference in abundance of histone peptides, which accounted for 14 and 36% of the total quantity of identified peptides in cryomilled and manually ground samples, respectively (**Figure 4B**). Ribosomal proteins were identified as abundant co-extracting contaminating proteins in both sets of samples, but they were more prevalent in cryomilled samples (55 and just 13% of the total peptide precursor area corresponded to ribosomal peptides in cryomilled and manually ground samples, respectively; **Figure 4B**). However, further quantitative analysis showed that RAs (obtained using Skyline software) of seventeen and eight histone peptide forms from N-termini of histones H3 and H4 (aa sequence regions 9–40 and 4–17, respectively), were very similar in samples prepared by the two techniques, despite the difference in purity of histone extracts (**Figure 4C**). Next, inter-sample variability related to the homogenization technique was evaluated using the same data set. The difference in mean log2-transformed peptide precursor areas obtained from analyses of samples prepared by the two grinding techniques was not significant (*p* = 0.271), but standard deviations of cryomilled samples were significantly higher than those of manually ground samples (*p* < 0.001; **Figure 4D**). Moreover, mean precursor areas were highly correlated, but not their standard deviations (Spearman correlation coefficients, 0.972 and -0.095, respectively). Thus, the manual homogenization treatment provided more suitable samples for mass spectrometric analysis of the *Arabidopsis* histone modifications in terms of both the histone sample purity and level of detected inter-sample variability.

As mentioned above, proportions of combinatorially modified peptide forms identified using protocols involving both types of homogenization were similar (**Figure 4C**), indicating that the homogenization technique did not influence the distribution of histone forms within the samples. Observed peptides of histone H3 included nine forms of KSTGGKAPR (9–17), three of KQLATKAAR (18–26) and five of KSAPATGGVKKPHR (27–40). The most abundant form of KSTGGKAPR was the non-modified form, followed by the form dimethylated on the first lysine (K9) and acetylated on the second lysine (K14). Other peptide forms with one mark (K9ac, K9me, K9me3) and two marks (K9meK14ac, K9me2K14ac, K9me3K14ac) were much less abundant. Similarly, for KQLATKAAR the non-modified form and the form with the first lysine acetylated (K18) were the most abundant, while the doubly acetylated form (K18acK23ac) was less abundant. Forms with the first lysine (K27) mono- or di-methylated were the most abundant of KSAPATGGVKKPHR, followed by the K27me3 form. The K27meK36me2 and non-modified forms were detected at very low abundance. Observed peptides of histone H4 were eight forms of GKGGKGLGKGGAKR (4–17), the most abundant being the non-modified form followed by two monoacetylated forms (at K16 and K12), the doubly acetylated form K12acK16ac and triple acetylated form K8acK12acK16ac. K5acK12ac/K8acK12ac, K5acK8acK12ac and K5acK8acK12acK16ac forms were also detected at lower abundance.

## Discussion

Changes in epigenetic modifications of histones are related to numerous biological processes and thus warrant attention in various scientific investigations. The problem of genetic and epigenetic variability in *A. thaliana* ecotypes grown under different conditions (in various localities) has been frequently dealt with, e.g., in the scope of the project of 1001 *A. thaliana* genomes and epigenomes (The 1001 Genomes Consortium, 2016 and references therein). On the other hand, inter-individual variability in the scope of respective accession has been less frequently studied although this parameter is crucial for convincing evaluation of various sets of data including high throughput epigenetic analyses. Detailed knowledge on the variability present among plant representatives in the control group is essential to assess the significance of respective changes between analyzed groups. In this respect, experimental design and sample pre-processing/preparation are important steps of any scientific study, including analyses of histone epigenetic marks. Moreover, as already mentioned, MS-based approaches have major advantages for such analyses, but more knowledge of the partitioning between biological and technical variation in the results they provide is needed. Thus, here we addressed these types of variation in MS-based plant epigenetic analysis. More specifically, we investigated: the inter-individual variability of histone mark levels in two *A. thaliana* ecotypes grown from seeds collected from one individual and sets of individuals. We also assessed the numbers of biological and technical replicates needed for statistically rigorous quantitative proteomic analysis. In addition, we compared effects of two plant tissue homogenization techniques on the quality of histone extracts.

We found that two *A. thaliana* ecotypes that are frequently used for diverse epigenetic analyses (Col-0 and Ws) had apparently different inter-individual variability in levels of histone peptide forms. Both comparison of aggregated technical replicates and variance decomposition by LMM showed that variance was very low in sets of Col-0 samples, even samples of plants grown from seeds collected from several parents (**Figures 1**, **2**). Thus, even leaves collected from plants originating from a heterogeneous mixture of seeds are sufficient for proteomic study of histone PTMs in Col-0, as the number of replicates needed is the same as for progenies of a single parent plant. In contrast, a significantly higher number of biological replicates was required for the Ws ecotype to reliably detect the same fold-changes in modified histone peptide abundance. Moreover, a significant difference in variances in histone peptide abundance was found between the Ws samples originating from seeds collected from a single individual and several parents (**Figure 2**). Thus, seeds collected from a single parent plant, are preferable for proteomic analysis of Ws due to the lower number of replicates needed. However, it should be noted that, in contrast to Col-0, an almost infeasibly large number of Ws plants (even originating from one parent plant) would be needed to identify less than 1.75-fold changes in levels of peptide forms with confidence (**Table 1**). These results may also reflect a higher level of so called developmental noise in the Ws ecotype, i.e., a higher level of fluctuations in protein expression and modifications. These fluctuations are the result of the intrinsically stochastic nature of molecular interactions that underlie transcription, translation and posttranslational regulation. The resulting cell-to-cell and inter-individual variations contribute to a rapid response to changes in the environment. An evolutionary tuning effect of this variation ensures the optimal fitness of a population (Novick and Weiner, 1957; Spudich and Koshland, 1976; Elowitz et al., 2002; Raser and O’Shea, 2004; Raj and van Oudenaarden, 2008; Eldar and Elowitz, 2010; Lestas et al., 2010; Chalancon et al., 2012). Next, higher variability in telomere lengths among representatives of the Ws ecotype compared to other *A. thaliana* ecotypes was observed. Ws telomeres displayed a bimodal size distribution, telomere tracts of the length 2 – 5 kb were found in some individuals and 4 – 9 kb in other plants (Shakirov and Shippen, 2004). Based on the obtained results, we encourage researchers to pay particular attention to the origin of studied organisms as the level of inter-sample variability might substantially differ, even between ecotypes of a single plant species. We recommend preliminary experimental assessment of biological variability of specimens of interest before beginning any comprehensive study, or at least application of recent advice to analyze more biological replicates when high biological variability is expected (Trutschel et al., 2015).

In our experiments, the technical variability related to LC-MS/MS analyses was found to contribute negligibly to the total variability. Accidentally, one technical batch was affected by emitter replacement and excluded from the data evaluation. Results of an LMM including data for the affected batch (presented in Supplementary Figure 3) show that factors such as an essential service intervention between runs may significantly reduce reproducibility, and analysis of an additional technical batch is warranted in such cases. Other factors that may contribute to a reduction in technical reproducibility have been identified in a recent intra-laboratory variability survey by the Protein Research Group of the Association of Biomolecular Resource Facilities (ABRF-PRG) (Bennett et al., 2015). Interestingly, a substantial association between preventative maintenance of the instrument prior to LC-MS/MS analyses and frequencies of outliers was reported, so the ABRF-PRG emphasized the need for thorough quality control after such events.

Proteomic-based histone characterization involves several steps (tissue homogenization, nuclei isolation, histone extraction, protein digestion, LC-MS/MS analysis and data processing). All of them are potentially bias-prone, but especially the tissue homogenization and protein extraction procedures (Piehowski et al., 2013). Typically, plant tissues are homogenized by blending or grinding in liquid nitrogen then extraction buffer is added to liberate nuclei for histone isolation. Such a time-consuming approach may distort analytical results in several ways, especially when applied to a large set of samples (Butt and Coorssen, 2006; Koroleva and Bindschedler, 2011). Thus, automation of plant tissue homogenization for histone extraction using a cryomill was introduced for fast, simple, efficient and reproducible homogenization of the samples. Surprisingly, however, the automated plant tissue homogenization procedure did not provide better quality extracts than manual grinding. Although both homogenization approaches led to identification of comparable set of histone proteins showing high reproducibility among the replicates, high numbers of other proteins were found in cryomilled samples. Increased protein complexity given especially by higher proportions of co-extracting ribosomal proteins subsequently affected detection of histone peptides during the MS/MS experiment. Similarly, histone extraction protocols designed to minimize handling of human cell culture samples were recently found to increase levels of co-extracted ribosomal proteins, but not to affect RA-based histone peptide quantification (Govaert et al., 2016). Accordingly, the RAs of selected histone peptide forms obtained from analyses of *A. thaliana* extracts prepared using our automated and manual homogenization procedures were similar, despite the difference in the purity of histone extracts. Nevertheless, our experience indicates that high absolute precursor peak areas are essential for identification, and especially quantification, of peptide forms with low abundance. Despite the reportedly higher reproducibility of automatic processing (Koroleva and Bindschedler, 2011), we observed higher inter-sample variability at quantitative level of histone peptides in cryomilled samples as evident by comparison between standard deviations of log2-transformed peptide precursor areas of samples ground in the cryomill and using a mortar and pestle. These observations altogether indicate that automatic processing albeit providing possible advantage for whole proteome analyses might not be beneficial for protein fractions or indicate a need for more thoroughly optimized automatic processing for a specific purpose. At least in case of histone extracts, data obtained corroborates the apparent superiority of manually grinding samples intended for mass spectrometric analysis of histone PTMs using a mortar and pestle.

Our results show that levels of combinatorially modified histone-peptide forms were comparable in *A. thaliana* Col-0 and Ws ecotypes (**Figure 1**). Unlike mixtures of truncated peptides identified after sample preparation using FASP alone, FASP followed by histone propionylation enabled correlation of the proportions of modified forms of peptide sequences with biological relevance (**Figure 4C**). In relatively abundant peptides with multiple epigenetic modifications all marks were acetylations, which are associated with open chromatin structure and high transcription levels. In agreement with published results on the co-localization of epigenetic marks corresponding to the specific chromatin state (Roudier et al., 2011; Sequeira-Mendes et al., 2014) relatively low level of peptide with co-occurrence of H3K9me2 (a modification typically associated with constitutive heterochromatin) and H3K14ac (euchromatin mark) was observed (**Figure 4C**).

Reporting of statistically significant results is a prerequisite for ensuring comparability of research outcomes. Our observations, together with previous findings, show that great care is needed in the design of proteomic experiments, including analysis of epigenetic marks, as diverse factors may affect apparently significant changes in peptide abundance. Regarding plant epigenetic studies, it might be assumed that even more care may be needed for analyses of plants that are not grown in tightly controlled environments – as, e.g., plants from field experiments. Thus, rigorous empirical evaluation of minimum required numbers of biological replicates is essential to acquire robust data.

## Author Contributions

SB cultivated plants and isolated histones. GL prepared samples for MS. DP performed LC-MS/MS analyses. GL evaluated MS data. II performed statistical analyses. ZZ, II, and GL were responsible for interpreting the proteomic data. MF and JF were responsible for interpreting the biological context. SB, MF, ZZ, II, and GL were responsible for writing the manuscript. All of the authors have read and approved the final version of the manuscript.

## Conflict of Interest Statement

The authors declare that the research was conducted in the absence of any commercial or financial relationships that could be construed as a potential conflict of interest.

## Footnotes

1 ftp://ftp.uniprot.org/pub/databases/uniprot/current_release/knowledgebase/reference_proteomes/Eukaryota/UP000006548_3702.fasta.gz

2 http://www.thegpm.org/crap/
