# Supplementary material for: Variations of Histone Modification Patterns: Contributions of Inter-plant Variability and Technical Factors
Source: Front Plant Sci. 2017 Dec 7;8:2084. doi: 10.3389/fpls.2017.02084 (PMC5725443; doi:10.3389/fpls.2017.02084)
Supplement: Supplementary file 1 [file Data_Sheet_1.pdf]

frontiers

Supplementary Material

Variations of histone modification pattern: contributions of inter-plant variability and technical factors

Sylva Brabencová[1,2], Ivana Ihnátová[1], David Potěšil[1], Miloslava Fojtová[1,2], Jiří Fajkus[1,2], Zbyněk Zdráhal[1,2], and Gabriela Lochmanová[1]*

*** Correspondence:** Gabriela Lochmanová: gabriela.lochmanova@ceitec.muni.cz

1. Supplementary Figures and Tables

1.1 Supplementary Figures

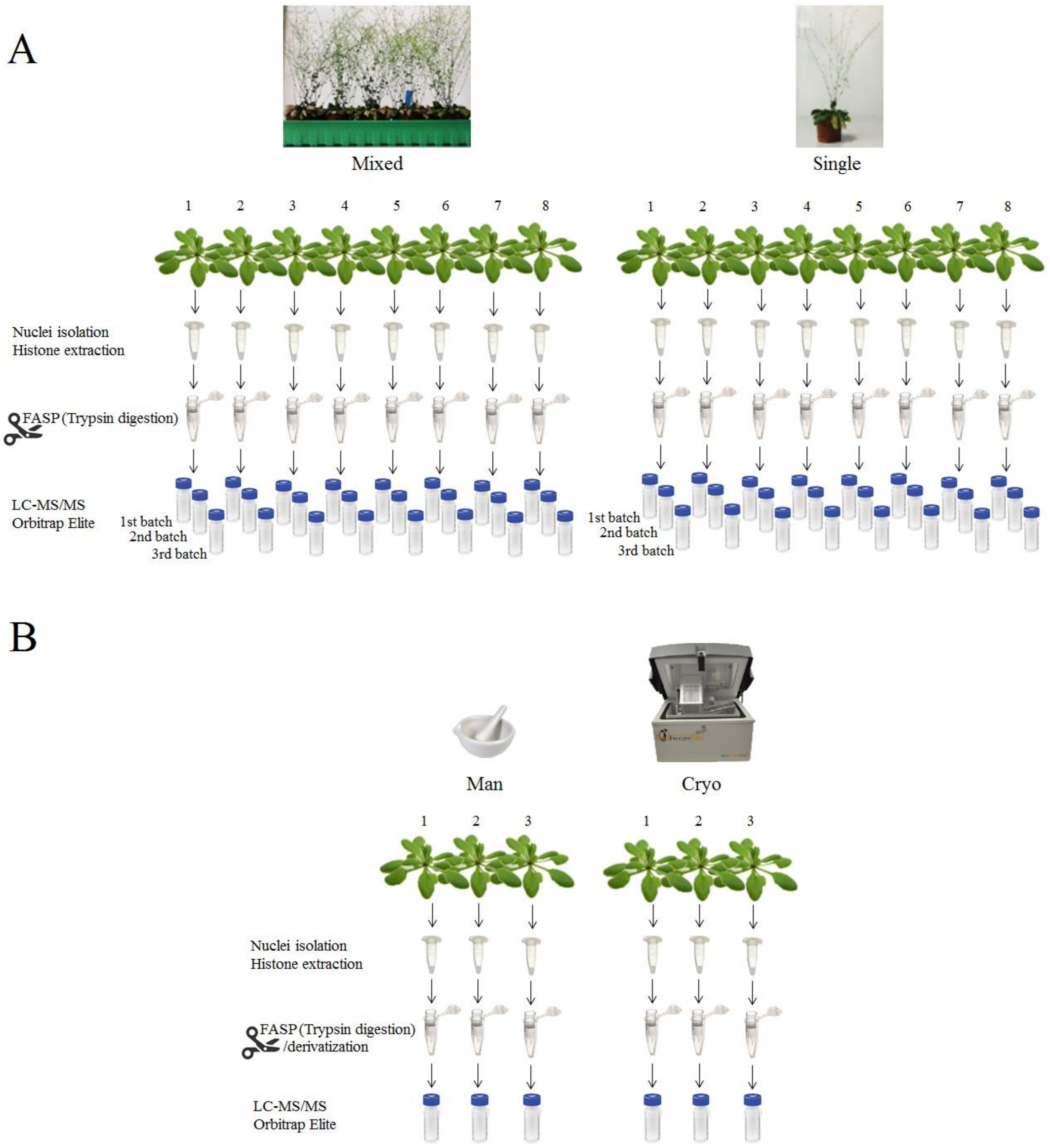

Supplementary Figure 1. Experimental design. **(A)** To assess inter-individual variability of histone mark levels in A. thaliana Col-0 and Ws ecotypes, histone proteins were extracted from leaves of independently cultivated plants growing from seeds collected from a single parent plant (Single) and from several simultaneously cultivated parent plants – progenies of different plants cultivated

independently (Mixed). Samples were prepared for analysis by FASP. Each group was represented by eight histone extracts (except Ws Single samples: seven extracts) which were analyzed in random order in three technical batches by MS. **(B)** To examine effects of the plant tissue homogenization technique, samples (biological triplicates) of independently cultivated Col-0 plants (Mixed) were homogenized using either a cryomill (Cryo) or a mortar and pestle (Man), then histone proteins were extracted, derivatized and digested with trypsin, and analyzed by MS.

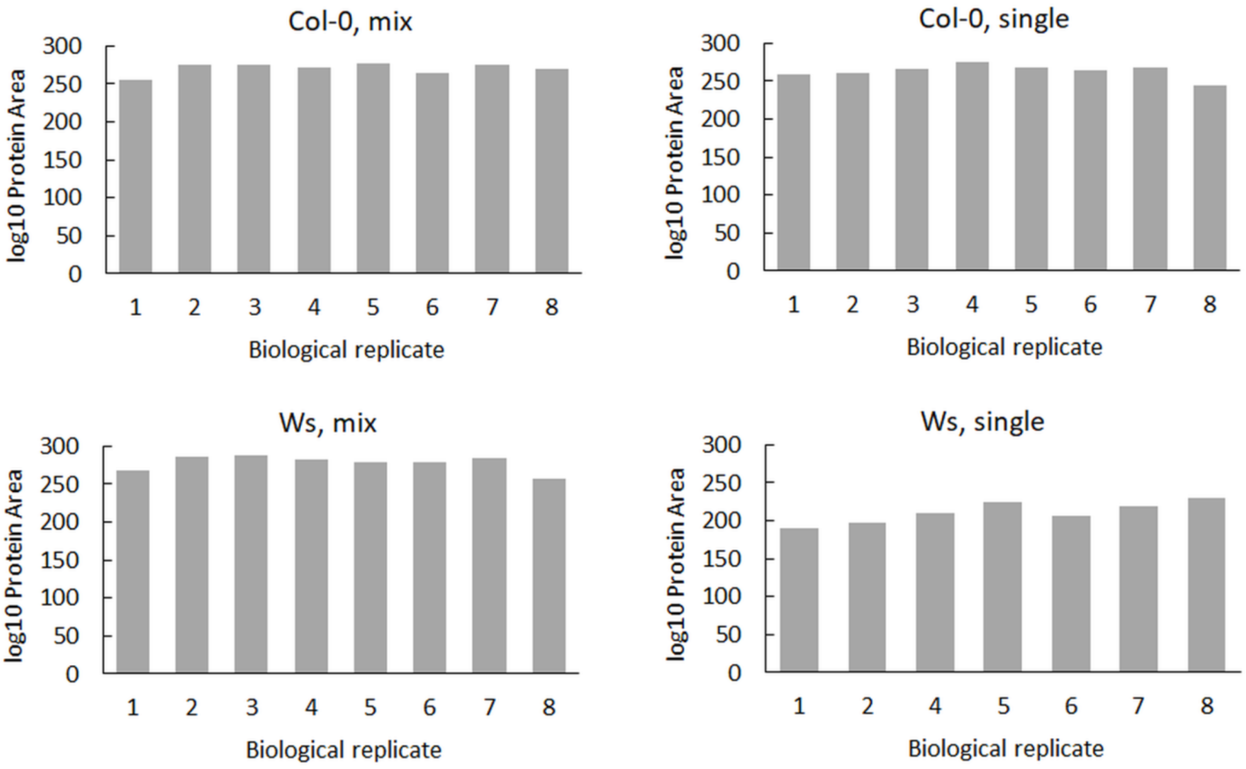

Supplementary Figure 2. Histone protein pool in individual biological replicates. Protein areas (obtained from Thermo Fisher Scientific; version 1.4) were log10-transformed and technical replicates were aggregated by medians. Values corresponding to histone proteins were summed to demonstrate that comparable histone pools between biological replicates were obtained when the same amount of starting material and homogenization technique were used.

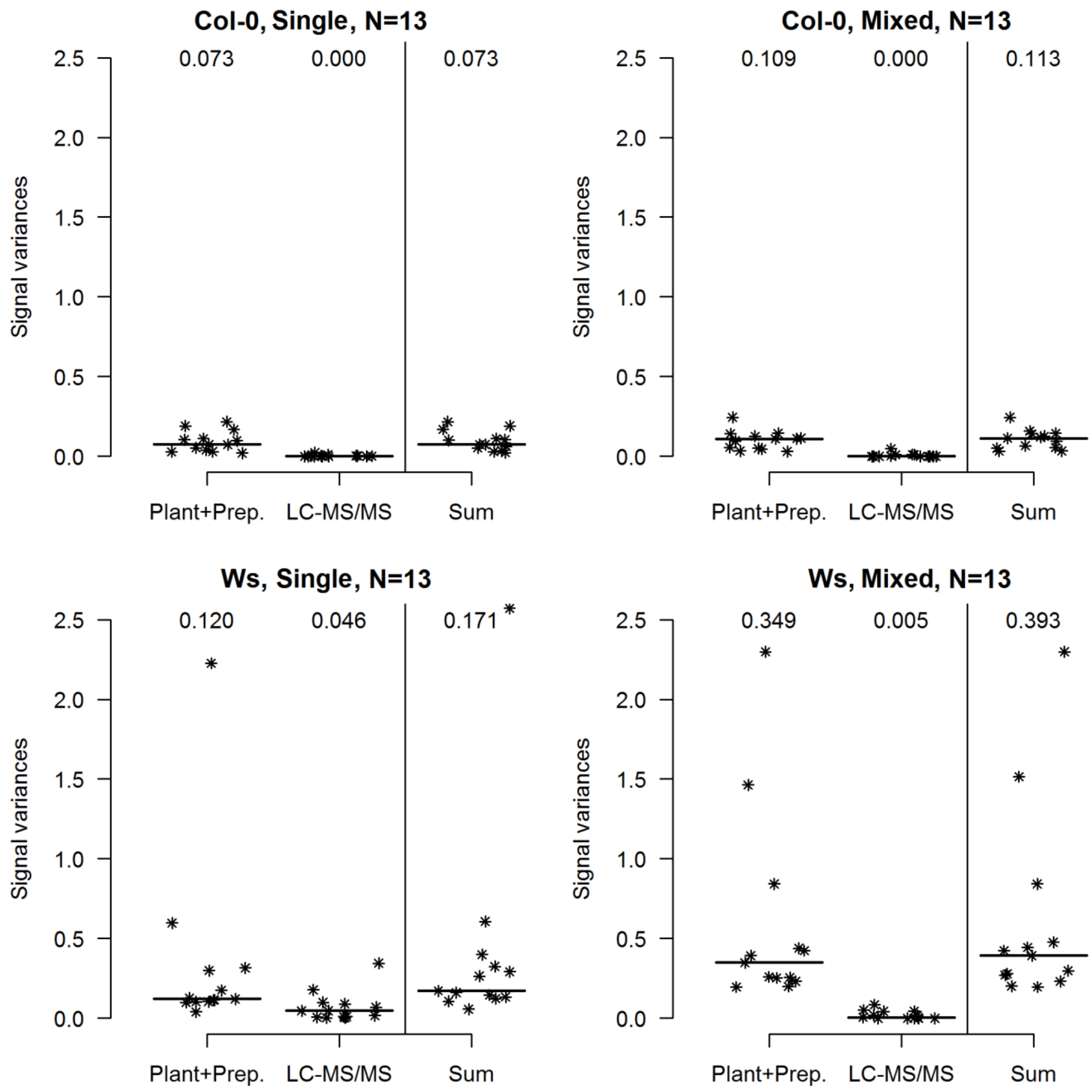

Supplementary Figure 3. Loss of reproducibility due to emitter failure during LC-MS/MS analysis of the first technical batch of *A. thaliana* Ws Single samples demonstrated by increased technical variance of histone mark levels (compare with Figure 2, which shows corresponding results, with data for the first technical batch of Ws Single samples excluded).

1.2 Supplementary Tables

Supplementary Table 1. Descriptive statistics – summarization of log2-transformed precursor areas of all biological replicates (following removal of outliers detected by the Grubbs test and averaging technical replicates).

	***A. thaliana* Col-0**							***A. thaliana* Ws**						
	**Mixed**			**Single**			**p-value***	**Mixed**			**Single**			**p-value***
	**N**	**Mean**	**SD**	**N**	**Mean**	**SD**		**N**	**Mean**	**SD**	**N**	**Mean**	**SD**	
**Histone H3**														
KSTGGK14acAPR/ STGGK14acAPR	8	28.09	0.19	8	28.18	0.23	0.653	8	27.90	1.17	7	26.75	0.52	0.064
K9acSTGGK14acAPR	8	25.37	0.11	8	25.46	0.21	0.137	8	26.47	0.8	7	25.48	0.29	0.027
KQLATK23acAAR/ QLATK23acAAR	8	26.45	0.26	8	26.30	0.23	0.698	8	24.87	1.17	7	24.32	1.47	0.571
K18acQLATK23acAAR	8	27.94	0.14	8	27.98	0.14	0.982	8	28.27	0.45	7	27.98	0.14	0.012
K27me1SAPATGGVK	8	28.58	0.29	8	28.54	0.23	0.549	8	28.88	0.27	7	28.19	0.23	0.750
K27me2SAPATGGVK	8	28.81	0.12	8	28.98	0.28	0.051	8	29.20	0.37	7	28.51	0.32	0.755
K27me3SAPATGGVK	8	27.14	0.19	8	27.10	0.34	0.155	8	27.81	0.44	7	27.11	0.21	0.095
**Histone H4**														
K12ac (aa 9-16)	8	25.55	0.28	8	25.63	0.21	0.463	8	24.95	0.42	7	24.43	0.48	0.742
K5acK8ac (aa 9-12)	8	24.75	0.18	8	24.79	0.11	0.208	8	24.71	0.43	7	23.91	0.23	0.141
K8acK12ac (aa 6-16)	8	23.24	0.25	8	23.31	0.16	0.244	8	22.88	0.56	7	22.55	0.22	0.034
K12acK16ac (aa 9-17)	8	27.01	0.08	8	27.02	0.11	0.480	8	26.73	0.32	7	26.02	0.23	0.443
K8acK12acK16ac (aa 6-17)	8	25.61	0.17	8	25.62	0.18	0.987	8	25.89	0.32	7	24.70	0.76	0.041
K5acK8acK12acK16ac (aa 4-17)	8	25.07	0.28	8	25.09	0.23	0.575	8	25.56	0.31	7	24.49	0.23	0.484
*p-value from F-test														

Supplementary Table 2. Protein concentration in independent replicates showing reproducibility of sample preparation before FASP. Although protein concentration was not measured in ecotype variability study, the data from preceding pilot studies are presented to show that comparable protein concentration is obtained when the same amount of starting material and homogenization technique are used. Aliquots of sulfuric extracts were sixteen times diluted with deionized water and protein content was determined using a Micro BCA™ Protein Assay Kit.

# Sample	Protein concentration (μg/ul)	Average protein concentration (μg/ul)	Standard deviation
1	0,09	0,13	0,04
2	0,17		
3	0,15		
4	0,09		
5	0,12		
6	0,16		
7	0,12		
8	0,19		
9	0,17		
10	0,16		
11	0,23		
12	0,13		
13	0,10		
14	0,15		
15	0,13		
16	0,14		
17	0,10		
18	0,12		
19	0,10		
20	0,13		
21	0,20		
22	0,19		
23	0,10		
24	0,11		
25	0,08		
26	0,10		
27	0,13		
28	0,16		
29	0,15		
30	0,13		
31	0,09		
32	0,10		
33	0,15		
34	0,14		
35	0,09		
36	0,09		
